# Spontaneous rupture and hemorrhage of a large simple hepatic cyst following external electrical muscle stimulation: a case managed by emergency laparoscopy

**DOI:** 10.1093/jscr/rjag209

**Published:** 2026-03-29

**Authors:** Wei Feng, Qingwang Ye, Qile Wang, Dongbo Zhao, Fanlai Meng

**Affiliations:** Department of Hepatobiliary and Pancreatic Surgery, The Affiliated Suqian Hospital of Xuzhou Medical University, No. 138, Huanghe South Road, Suqian, Jiangsu 223800, China; Department of Hepatobiliary and Pancreatic Surgery, The Affiliated Suqian Hospital of Xuzhou Medical University, No. 138, Huanghe South Road, Suqian, Jiangsu 223800, China; Department of Hepatobiliary and Pancreatic Surgery, The Affiliated Suqian Hospital of Xuzhou Medical University, No. 138, Huanghe South Road, Suqian, Jiangsu 223800, China; Department of Hepatobiliary and Pancreatic Surgery, The Affiliated Suqian Hospital of Xuzhou Medical University, No. 138, Huanghe South Road, Suqian, Jiangsu 223800, China; Department of Pathology, The Affiliated Suqian Hospital of Xuzhou Medical University, No. 138, Huanghe South Road, Suqian, Jiangsu 223800, China

**Keywords:** simple hepatic cyst, spontaneous rupture, electrical muscle stimulation, laparoscopic fenestration, povidone-iodine ablation

## Abstract

Hemorrhagic rupture of a simple liver cyst is a rare clinical occurrence. Whether external physical forces can trigger such rupture remains unclear. A 47-year-old woman presented with acute abdominal pain 72 h following an electrical muscle stimulation (EMS) session for weight reduction. Cross-sectional imaging demonstrated a 10-cm ruptured cyst in the right hepatic lobe with associated 1.5 l of hemoperitoneum. Her platelet count was 88 × 10^9^/l. The patient successfully underwent emergency laparoscopic cyst deroofing with povidone-iodine sclerotherapy and did not require blood transfusion. Histopathology confirmed a simple biliary cyst, and a follow-up magnetic resonance imaging at 6 months showed no recurrence. This case suggests that vigorous muscular contractions from EMS may act as a potential precipitating factor for the rupture of large liver cysts, particularly in the context of mild thrombocytopenia, while spontaneous rupture cannot be entirely excluded as an alternative etiology. Laparoscopic management with chemical ablation represents an effective emergency treatment strategy.

## Introduction

Simple hepatic cysts (SHCs) are frequently encountered benign liver lesions. Their epidemiology is well-summarized in recent guidelines, including the 2023 EASL clinical practice update [1]. Cyst rupture is an uncommon complication [2]. A review of the literature reveals only 10 reported cases, half of which involved significant hemorrhage [3]. Most events were described as spontaneous, with very few linked to trauma or medical procedures. An unresolved question is whether external mechanical forces—such as those generated during abdominal muscle stimulation—can produce a sufficient pressure differential to cause cyst rupture. This case provides a clinical context to explore this potential mechanism.

## Case presentation

A 47-year-old woman was evaluated in the emergency department for acute, severe pain in the right upper quadrant. Three days earlier, she had completed a 30-min session of low-frequency electrical muscle stimulation (EMS) aimed at weight loss, tolerating the procedure without immediate discomfort.

On admission, her vital signs were stable. Physical examination revealed tenderness in the right upper abdomen without guarding or rebound. Laboratory tests showed a platelet count of 88 × 10^9^/l; other parameters were within normal limits.

**Figure 1:**
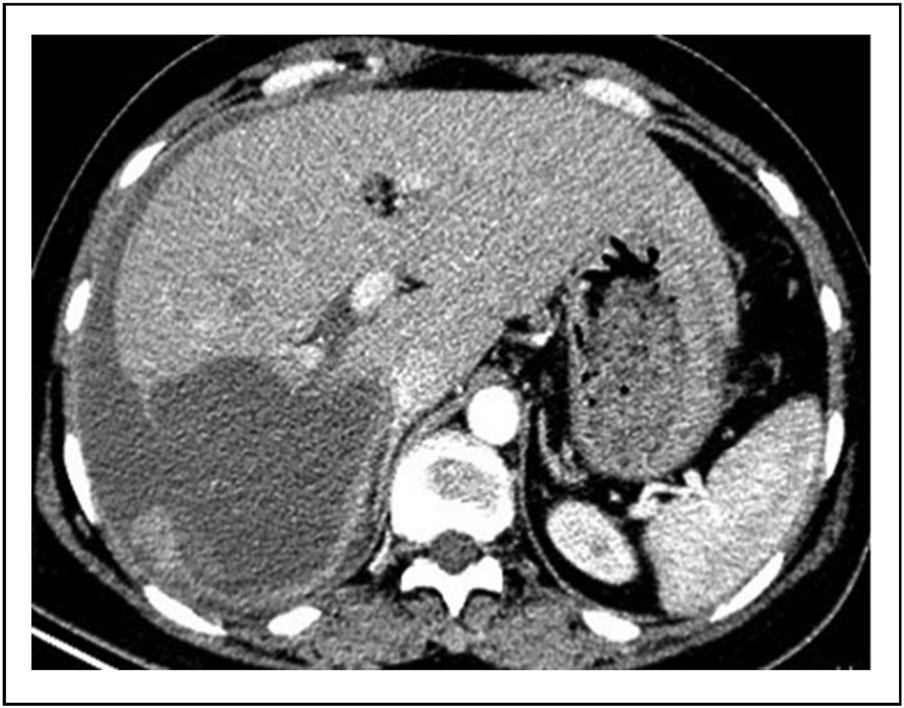
Contrast-enhanced CT image showing the ruptured right hepatic lobe cyst and surrounding hemoperitoneum.

**Figure 2:**
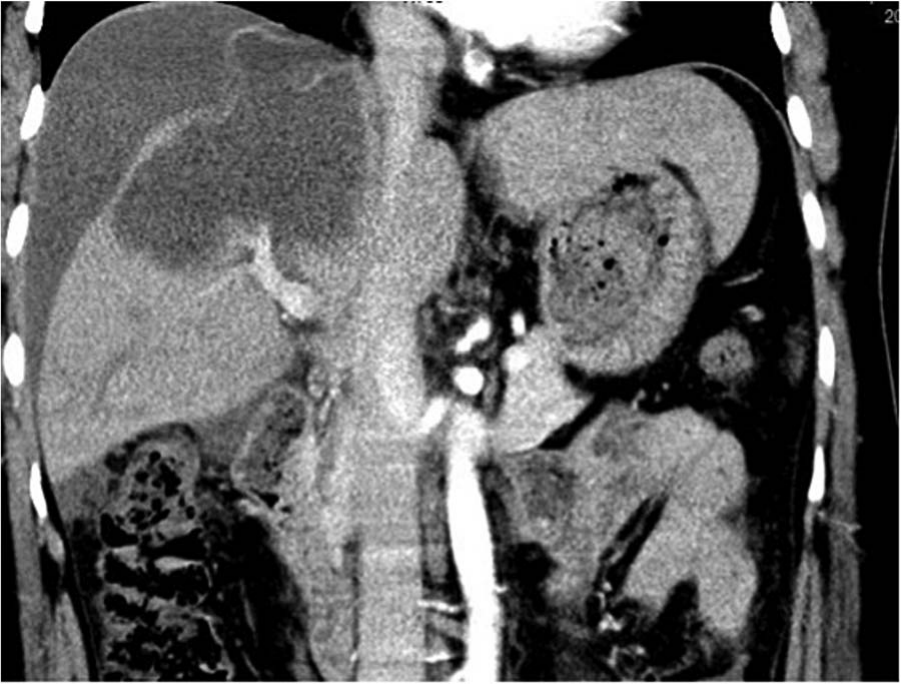
Contrast-enhanced CT image showing the ruptured right hepatic lobe cyst and surrounding hemoperitoneum.

Contrast-enhanced abdominal computed tomography (CT) demonstrated a 10-cm cystic lesion in the right hepatic lobe with a disrupted wall, linear enhancement of the adjacent liver capsule, and a large volume of free intraperitoneal fluid (~1.5 l) (Figs 1 and 2). Diagnostic paracentesis yielded non-clotting bloody fluid.

Emergency laparoscopic exploration confirmed ~1500 ml of hemoperitoneum and an actively bleeding, ruptured cyst on the anterior surface of the right hepatic lobe. Laparoscopic deroofing was performed using an ultrasonic knife, extending to the parenchymal edge. The remaining epithelial lining was then treated with 10% povidone-iodine for chemical ablation. A drain was placed post-procedure. Total operative time was 110 min with an estimated blood loss of <10 ml.

**Figure 3:**
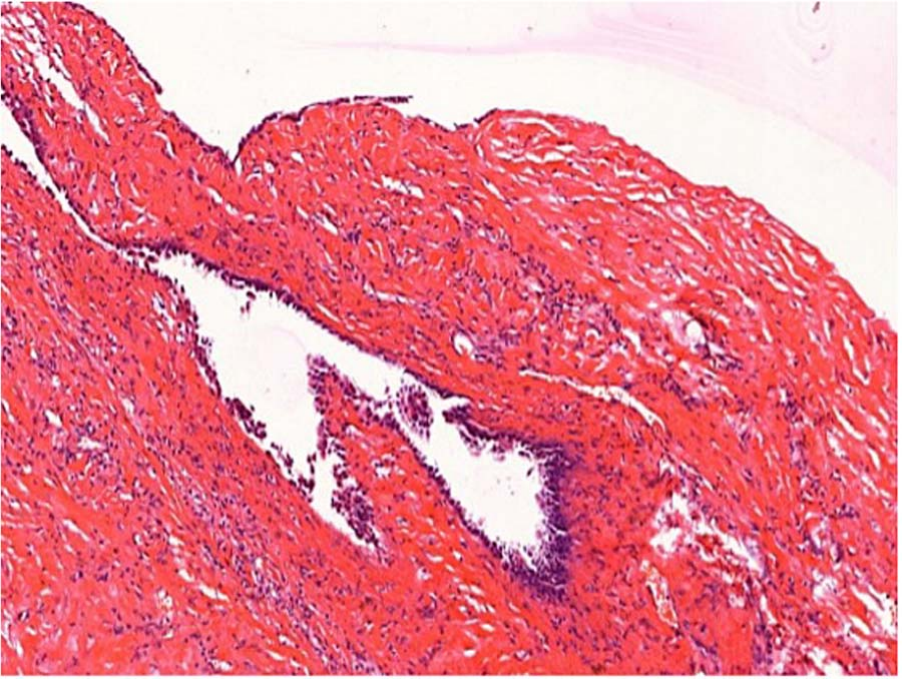
Histopathology (H&E staining, ×200) confirming a simple biliary cyst without features of hydatid disease.

**Figure 4:**
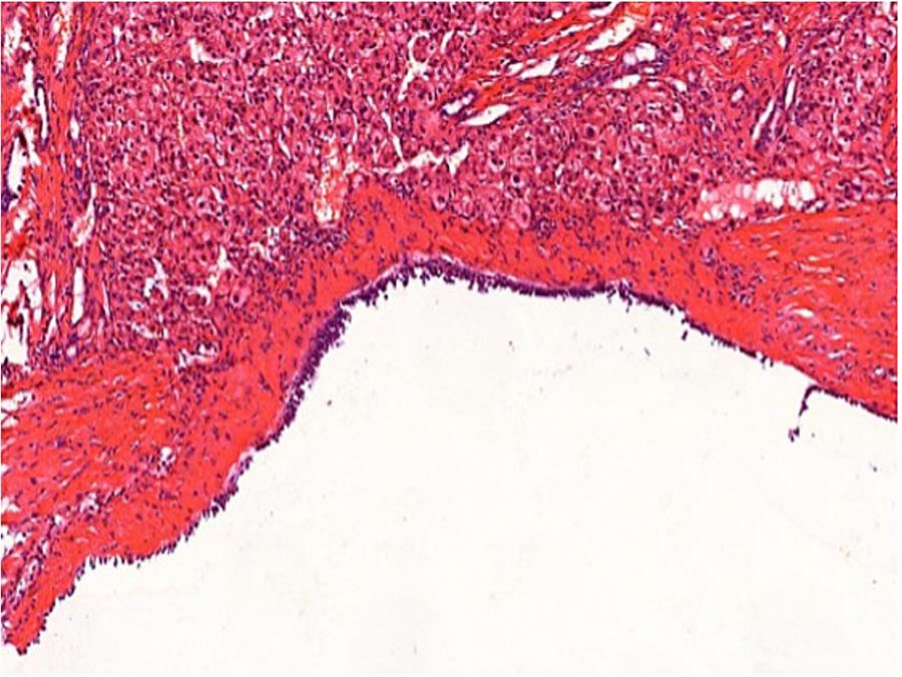
Histopathology (H&E staining, ×200) confirming a simple biliary cyst without features of hydatid disease.

Histopathological examination confirmed a simple biliary cyst with no evidence of parasitic infection or malignancy (Figs 3 and 4). The postoperative course was uneventful, and the patient was discharged on the third day. Follow-up magnetic resonance imaging (MRI) at 6 months showed no recurrence. (Table 1).

**Table 1:** Clinical timeline.

Time point	Event	Key details
3 days pre-rupture	EMS training	The patient did an EMS training session that lasted 30 min. Low-frequency electrical stimulation was used, and the patient had no discomfort during or after the training
Day 0, 08:00	Sudden abdominal pain	The patient felt acute colic in the right upper abdomen, and this pain lasted for 2 h
Day 0, 08:30	Emergency department visit	Vital signs were checked: heart rate was 91 beats per minute, blood pressure was 116/63 mmHg. Tenderness was found when pressing the right upper abdomen
Day 0, 09:15	Imaging test	An abdominal CT scan was done. It showed a 10 cm ruptured cyst in the right liver, along with 1.5 l of ascites in the abdominal cavity
Day 0, 10:00	Diagnostic abdominal paracentesis	20 ml of non-clotting blood was drawn through paracentesis, confirming internal bleeding in the abdomen
Day 0, 11:30	Surgery started	The surgical plan was laparoscopic cyst unroofing, combined with povidone-iodine chemical ablation for treatment
Day 0, 13:20	Surgery completed	The entire surgery took 110 min. Intraoperative blood loss was less than 10 ml, and the procedure went smoothly without any issues
Day 3 post-onset	Discharge from hospital	The patient recovered well after surgery with no complications. A 6-month follow-up MRI scan showed negative results, indicating a successful recovery with no recurrence

## Discussion

Mechanistically, the tonic abdominal contraction induced by EMS can generate a transient increase in intra-abdominal pressure, estimated at 12–15 mmHg based on recent biomechanical models [4]. In the context of a large cyst exceeding 8 cm, Laplace’s law dictates that wall tension is already approaching a critical threshold [5]. The pressure wave from EMS may therefore have acted as a potential precipitating event for rupture, though this causal link remains speculative. To our knowledge, this represents the first reported association between EMS and SHC rupture, and spontaneous rupture cannot be ruled out as the primary cause in this case. Future studies utilizing computational modeling could help quantify the precise pressure dynamics involved and clarify whether a causal relationship exists. The patient presented with a platelet count of 88 × 10^9^/l, which is below the conventional lower limit. Available evidence indicates that

a platelet count below 100 × 10^9^/l is associated with an increased risk of bleeding after hepatic procedures (odds ratio (OR) ~ 2.41) [6, 7]. In patients with large cysts, such thrombocytopenia may impair the hemostatic sealing of minor capsular breaches, thereby potentiating the risk of significant hemoperitoneum upon rupture. While a small randomized controlled trial (RCT) in elective settings demonstrated that povidone-iodine ablation reduces 5-year recurrence from 31% to 8%, no analogous data exist for the emergency management of ruptured cysts [8, 9]. Given the favorable outcome in this case, the adjunctive use of chemical ablation appears clinically reasonable for the emergency management of ruptured SHCs. Establishing a prospective registry to track recurrence and complication rates specifically in ruptured cases would be valuable for developing evidence-based guidelines for this rare clinical scenario. Including the present case, a total of 18 cases of ruptured SLC have been reported in the literature since 1970, and hemorrhage complicated 17 of these cases (94.4%), with 16 requiring surgical intervention [10–17]. While prior cases have been attributed to trauma, spontaneous rupture, or iatrogenic causes [11], this case expands the spectrum of potential triggers by proposing EMS as a plausible contributor rather than a definitive causal factor, and highlights the need for further investigation into the potential association between external abdominal muscle stimulation and hepatic cyst rupture.

## Conclusion

EMS may act as a potential precipitating factor for the rupture of large SHCs, particularly in the setting of thrombocytopenia. Laparoscopic deroofing combined with iodine ablation offers a safe and effective emergency treatment. Further studies should aim to identify cyst size and platelet thresholds that might guide preventive measures for patients with large hepatic cysts undergoing external physical interventions.

## References

[1] European Association for the Study of the Liver (EASL) . EASL clinical practice guidelines on the management of benign liver tumours. J Hepatol 2016;65:386–98. 10.1016/j.jhep.2016.04.001 27085809

[2] Lantinga MA, Gevers TJ, Drenth JP. Evaluation of hepatic cystic lesions. World J Gastroenterol 2013;19:3543–54. 10.3748/wjg.v19.i23.3543 23801855 PMC3691048

[3] Vannucchi A, Masi A, Vestrini G et al. Extraperitoneal hemorrhagic rupture of a simple hepatic cyst. A case report and literature review. Ann Ital Chir 2016a;87:S2239253X16025883. 27734812

[4] Neumann P, Gill V. Pelvic floor and abdominal muscle interaction: EMG activity and intra-abdominal pressure. Int Urogynecol J Pelvic Floor Dysfunct 2002;13:125–32. 10.1007/s001920200027 12054180

[5] Della Corte M, Viggiano D. Wall tension and tubular resistance in kidney cystic conditions. Biomedicines 2023;11:1750. 10.3390/biomedicines11061750 37371845 PMC10296710

[6] Wang X, Wang P, Tang B et al. Platelet count as a double-edged sword: the impact of thrombocytosis and thrombocytopenia on long-term outcomes after hepatic resection for hepatocellular carcinoma. Biosci Trends 2025;19:351–60. 10.5582/bst.2025.01067 40268417

[7] Northup PG, Garcia-Pagan JC, Garcia-Tsao G et al. Vascular liver disorders, portal vein thrombosis, and procedural bleeding in patients with liver disease: 2020 practice guidance by the American Association for the Study of Liver Diseases. Hepatology 2021;73:366–413. 10.1002/hep.31646 33219529

[8] E vS, Wroblicka JT, HB D'A et al. Symptomatic hepatic cysts: percutaneous drainage and sclerosis. Radiology 1994;190:387–92. 10.1148/radiology.190.2.8284385 8284385

[9] Lantinga MA, Gevers TJ, Drenth JP. Evaluation of hepatic cystic lesions. World J Gastroenterol 2013;19:3543–54. 10.3748/wjg.v19.i23.3543 23801855 PMC3691048

[10] Vannucchi A, Masi A, Vestrini G et al. Extraperitoneal hemorrhagic rupture of a simple hepatic cyst. A case report and literature review. Ann Ital Chir 2016b;87:S2239253X16025883. 27734812

[11] Park J . Traumatic rupture of a non-parasitic simple hepatic cyst presenting as an acute surgical abdomen: case report. Int J Surg Case Rep 2019;65:87–90. 10.1016/j.ijscr.2019.10.051 31698200 PMC6920217

[12] Amaral MJ, Serôdio M, Koch MJ et al. Ruptured hemorrhagic hepatic cyst: an unusual case report. GE Port J Gastroenterol 2020;27:124–7. 10.1159/000502359 32266310 PMC7113593

[13] Imagami T, Takayama S, Maeda Y et al. Transcatheter arterial embolization for hemorrhagic rupture of a simple hepatic cyst: a case report. Radiol Case Rep 2021;16:1956–60. 10.1016/j.radcr.2021.04.066 34149982 PMC8193074

[14] Mesh A, Thomas C, Nanayakkara G et al. Conservative management of a ruptured simple hepatic cyst: a case report. Cureus 2025; 17:e97594. 10.7759/cureus.97594 41450364 PMC12728242

[15] Shimada S, Hara Y, Wada N et al. Spontaneously ruptured hepatic cyst treated with laparoscopic deroofing and cystobiliary communication closure: a case report. Asian J Endosc Surg 2016;9:208–10. 10.1111/ases.12284 27120973

[16] Imaoka Y, Ohira M, Kobayashi T et al. Elective laparoscopic deroofing to treat the spontaneous rupture of a large simple liver cyst: a case report. Surg Case Rep 2016;2:148. 10.1186/s40792-016-0275-x 27928780 PMC5143329

[17] Karasuyama T, Kiguchi G, Masui H et al. Laparoscopic hepatic cyst fenestration using linear staplers for a ruptured recurrent hepatic cyst: a case report. Asian J Endosc Surg 2025;18:e70100. 10.1111/ases.70100 40523658

